# Maternal vitamin D status during pregnancy and infant’s gut microbiota: a prospective cohort study

**DOI:** 10.3389/fnut.2024.1428356

**Published:** 2024-07-29

**Authors:** Qiying Song, Yao Li, Tao Zhou, Meiqun Xiao, Bin Xiao, Mengying Wang, Yuanfang Zhu

**Affiliations:** ^1^Department of Child Healthcare, Shenzhen Baoan Women's and Children's Hospital, Shenzhen, China; ^2^Department of Obstetrics, Shenzhen Baoan Women's and Children's Hospital, Shenzhen, China; ^3^Department of Epidemiology and Biostatistics, School of Public Health (Shenzhen), Shenzhen Campus of Sun Yat-sen University, Shenzhen, China; ^4^Maternal-Fetal Medicine Institute, Shenzhen Baoan Women's and Children's Hospital, Shenzhen, China; ^5^Department of Nutrition and Food Hygiene, School of Public Health, Peking University, Beijing, China; ^6^Key Laboratory of Epidemiology of Major Diseases (Peking University), Ministry of Education, Beijing, China

**Keywords:** serum 25(OH)D level, maternal, pregnancy, gut microbiota, dynamic change, infant

## Abstract

**Objectives:**

To prospectively explore the association of maternal serum 25(OH)D levels with the infant’s gut microbiota in Chinese populations, and to evaluate its potential influence on the dynamic change patterns of offspring’s gut microbiota from 1 to 6 months old.

**Methods:**

Eighty-seven mother-infant dyads (vitamin D insufficient group vs. normal group = 59 vs. 28) were included in this longitudinal study. Two fecal samples were collected for the included infant at home by the parents at 1 month of age (“M1 phase”) and 6 months of age (“M6 phase”). Gut microbiota were profiled by 16S rRNA gene sequencing. We performed mixed effects models on alpha diversity metrics, PERMANOVA tests on beta diversity distances, and linear discriminant analysis (LDA) to identify differently abundant taxa.

**Results:**

We observed significantly lower Pielou’s evenness and Shannon diversity in the vitamin D insufficient group in the M6 phase (*p* = 0.049 and 0.015, respectively), but not in the M1 phase (*p* > 0.05), and the dynamic changes in alpha diversity from 1 to 6 months old were significantly different according to maternal vitamin D status (*p* < 0.05). There were also significant differences in gut microbiota composition between the vitamin D insufficient group and normal group, both in the M1 and M6 phases (LDA score > 2.0, *p* < 0.05). Moreover, among the predicted metagenome functions, pathways related to amino acid biosynthesis, starch degradation, and purine nucleotides biosynthesis were enriched in the vitamin D insufficient group.

**Conclusion:**

Our findings highlight that maternal vitamin D status plays a pivotal role in shaping the early-life gut microbiota of the next generation.

## 1 Introduction

A growing body of evidence suggests that vitamin D during pregnancy is essential for optimal maternal and offspring health (1–3). Maternal vitamin D deficiency or insufficiency was considered as a significant risk factor for adverse maternal-fetal outcomes, including preeclampsia, gestational diabetes mellitus, low birth weight, and preterm births (2).

It is well acknowledged that early life gut microbiota is influenced by various factors during pregnancy, such as maternal antibiotic and probiotic uses, dietary intake, and diseases of pregnancy (4–6). In recent years, the role of vitamin D during pregnancy in the infant’s gut microbiota has been of increasing interest. In vitamin D receptor knockout mice, there were significant communal and functional changes in the gut microbiota compared with wild type mice, the mechanism of which may be related to reduced inflammation, upregulation of innate immunity, etc. (7–9). The latest systematic review research has summarized associations between maternal vitamin D supplementation during pregnancy and infant’s gut microbiota, and revealed that maternal supplementation with vitamin D could impact the gut microbiota composition of offspring (10). However, this systematic review included only eight articles (two animal studies, three randomized clinical trials, and three cohort studies), and the large heterogeneity of the results prevented them from drawing a reliable conclusion.

In addition to vitamin D supplementation, only a few studies focused on vitamin D levels in the blood. In the KOALA birth cohort, Talsness et al. (11) found that maternal plasma vitamin D levels, which were determined at approximately the 36th week of pregnancy, were associated with counts of several limited bacterial taxa of infants at 1 month of age. Cord blood vitamin D was associated with increased *Lachnobacterium* and decreased *Lactococcus* in the Vitamin D Antenatal Asthma Reduction Trial (12). Another recent study reported that higher prenatal plasma and cord 25(OH)D levels were significantly associated with lower richness and diversity of the gut microbiota of their offspring at 1 month of age (13). These findings suggest maternal vitamin D levels might influence the infant’s gut microbiota composition and structure. However, questions remain and there are no clear conclusions. Moreover, most of previous studies were conducted in European and American populations, studies on Chinese populations were limited. Due to possible racial differences, studies conducted in Chinese populations are needed.

Therefore, in the present study, we aimed to explore the association of maternal vitamin D levels during pregnancy with the infant gut microbiota in a prospective cohort study in Shenzhen, China. In addition, we would evaluate the potential influence of maternal vitamin D status on the dynamic change patterns of offspring’s gut microbiota from 1 to 6 months old for the first time.

## 2 Methods

### 2.1 Participants

The present study was conducted at the Shenzhen Baoan Women’s and Children’s Hospital from November 2020 to December 2021, and was approved by the Ethical Committees of Shenzhen Baoan Women’s and Children’s Hospital (approval number: LLSC 2020-09-02-KS). All participants provided their written informed consents, and all methods were carried out in accordance with relevant guidelines.

Women enrolled in this study were between 20 and 45 years old, in their second trimester, with serum 25(OH)D measured, and living in Shenzhen. Those who would not possibly give birth at the Shenzhen Baoan Women’s and Children’s Hospital were excluded. A total of 87 mother-infant dyads were included in the present study, with all infants born of a singleton pregnancy with full-term (≥37 weeks and <42 weeks of gestation) and normal birth weight (≥2,500 g and ≤4,000 g). Newborns with any obvious congenital abnormality, neurological dysfunction, fetal chromosomal abnormality, or metabolic diseases were excluded. None of the infants received antibiotics or probiotics before the 6-month visit.

Information on maternal and infant characteristics, including maternal age at delivery, pre-pregnancy body mass index (BMI), gestational weight gain, gestational age, delivery mode, gestational diabetes mellitus (GDM), hypertensive disorders of pregnancy (HDP), infant’s sex, birth weight and length were derived from the electronic medical records of the hospital information system. In addition, information on feeding practices (breastfeeding, formula, or mixed), weight, length, and head circumference at 1 month and 6 months of age were also collected from the medical records.

### 2.2 Maternal serum vitamin D measurement

Maternal 25(OH)D levels were measured in serum samples from the second trimester of pregnancy (median = 17 weeks, range: 15 ~ 19 weeks) in the laboratory of Shenzhen Baoan Women’s and Children’s Hospital using chemiluminescence immunoassay (CLIA) technology. There is no consensus on optimal vitamin D levels during pregnancy. In the present study, 25(OH)D < 30 ng/mL is considered to be vitamin D insufficiency, whereas 25(OH)D ≥ 30 ng/mL is considered normal levels (3, 14).

### 2.3 Collection of fecal samples

Two fecal samples were collected for the included infants at home by the parents at 1 month of age (median = 30 days, range: 26 ~ 42 days, henceforward referred to as “M1 phase”) and again at 6 months of age (median = 184 days, range: 170 ~ 258 days, henceforward referred to as “M6 phase”). All infants had no fever, diarrhea, or other symptoms during the week prior to sampling. All fecal samples were collected using sterile tubes and temporarily placed in ice boxes and then carried or mailed to the hospital, and finally stored in a −80°C freezer within 24 h until DNA extraction.

### 2.4 Sequencing and sequence processing

Genomic DNA was extracted from fecal samples using the MoBio PowerSoil DNA isolation kit (MoBio, Carlsbad, CA) according to the manufacturer’s instructions. The concentration and quality were assessed using Qubit (Invitrogen) and verified by agarose gel electrophoresis. The 16S rRNA gene was amplified using 338F/806R primer pair (forward primer: 5′-ACTCCTACGGGAGGCAGCAG-3′, and reverse primers: 5′-GGACTACHVGGGTWTCTAAT-3′), targeting the V3-V4 hypervariable regions. All quantified amplicons were equally pooled and sequenced on the Illumina MiSeq system (Illumina Inc., CA, United States) with the paired-end mode. The raw sequencing data were deposited into the China National GeneBank DataBase (CNGBdb Project ID: CNP0005435).

The 16S rRNA gene sequences were analyzed using QIIME2 software (version 2020.11) with the following steps (15). Firstly, the raw sequencing reads were demultiplexed with a custom Perl script, and then the paired-end sequencing reads were imported into a QIIME2 artifact with the command “qiime tool import.” Secondly, quality filtering was followed, including the removal of Phix and the processing of chimeric sequences with the command “qiime dada2 denoise-paired.” Thirdly, run the command “qiime2 feature-classifier classify-sklearn” against the Greengenes (13_8 revision) database to finish the taxonomic assignment (16). Finally, the indexes of alpha and beta diversity were generated with the command “qiime phylogeny align-to-tree-mafft-fasttree” and “qiime diversity core-metrics-phylogenetic,” respectively, at a sample depth of 10,000 according to the tutorials of QIIME2 (15).

### 2.5 Statistical analysis

Statistical analysis was performed using R software (version 3.6.1). A *p*-value < 0.05 was considered statistically significant for all tests.

#### 2.5.1 General characteristics comparison

To compare the differences in general characteristics between the vitamin D insufficient group and normal group, continuous characteristics were analyzed using unpaired *t*-tests and reported as means ± standard deviation (SD), while categorical data were studied with the chi-square test or Fisher’s exact tests and presented as numbers and percentages.

#### 2.5.2 Alpha and beta diversity

Alpha diversity metrics refer to the diversity within a particular area or ecosystem, summarizing the structure of an ecological community concerning its richness (number of taxonomic groups, e.g., observed feature value), evenness (distribution of abundances of the groups, e.g., Pielou’s evenness index), or both (e.g., Shannon diversity index) (17). Wilcoxon rank sum tests were applied to compare the above mentioned three alpha diversity metrics between the vitamin D insufficient group and normal group. Linear mixed effects models were performed to evaluate the changes in alpha diversity from the M1 to M6 phase, adjusted for maternal pre-pregnancy BMI, gestational weight gain, gestational age, delivery mode, infant’s sex, and feeding practices, with a random effect of subjects.

Beta diversity quantifies differences in the overall taxonomic composition between two samples or ecosystems, commonly using the “distance” to capture the dissimilarity (18). In the present study, Bray-Curtis distance was applied to determine multivariate sample distances and visualized by principal coordinates analysis (PCoA). Permutational Multivariate Analysis of Variance (PERMANOVA) was used to compute the cross-sectional difference of beta diversity between the vitamin D insufficient group and normal group in the M1 and M6 phases. To test for the differential change in beta diversity from the M1 to M6 phase in offspring born to mothers with and without vitamin D insufficiency, a PERMANOVA model was fitted with a two-way interaction between vitamin D status and time. All analyses were adjusted for maternal pre-pregnancy BMI, gestational weight gain, gestational age, delivery mode, infant’s sex, and feeding practices.

#### 2.5.3 Relative abundance analyses

At various taxonomic levels (from phylum to genus level), linear discriminant analysis (LDA) was performed, and the LDA Effect Size (LEfSe) with the logarithmic LDA score threshold set at 2.0 was applied to identify taxonomic biomarkers that characterize the differences between the vitamin D insufficient group and normal group (19).

#### 2.5.4 Abundant pathways and ecological association networks analyses

In addition, pathway analysis for metabolomics data was conducted to predict enriched pathway of differential metabolites. PICRUSt2.0 (Phylogenetic Investigation of Communities by Reconstruction of Unobserved States) in QIIME2 was used to predict microbiome function based on the Greengenes 16S rRNA database and KEGG orthologs (20). The different metabolic functional pathways between the vitamin D insufficient group and normal group were generated using the STAMP (v2.1.3) program (21), followed by Benjamini-Hochberg correction.

What’s more, regarding that gut bacteria usually do not exist independently, but interact with each other and form a dynamic network that may significantly impact human health, determining networks of microbial interactions is important for the functional characterization of a microbial community. Therefore, SPIEC-EASI (SParse InversE Covariance Estimation for Ecological ASsociation Inference), a statistical method for the inference of microbial ecological networks from amplicon sequencing datasets, was used to analyze the microbial ecological network (22).

## 3 Results

### 3.1 Cohort characteristics

Eighty-seven infants (vitamin D insufficient group vs. normal group = 59 vs. 28) were included in the present study, with two visits at 1 and 6 months of age, respectively. In general, maternal age at delivery was around 31 years old, and gestational age was around 39.3 weeks, with an average pre-pregnancy BMI of 21 kg/m^2^ and gestational weight gain of 14.5 kg. About 40 percent of the mothers were diagnosed with GDM, 35 percent of the infants were born by cesarean section, and about 70 percent were breastfed. Table 1 shows the maternal and infant characteristics comparison between the vitamin D insufficient group and normal group. Except for maternal serum vitamin D level, which was significantly lower in the vitamin D insufficient group (22.1 ± 4.5 vs. 33.2 ± 1.5 ng/mL, *p* < 0.001), there were no significant differences between the two groups, signifying comparable general characteristics.

**Table 1:** Maternal and infant characteristics comparison between the vitamin D insufficient group and normal group (*N* = 87).

	Vitamin D status		*p* value
	Insufficient group (*N* = 59)	Normal group (*N* = 28)	
**Maternal characteristics**			
Age at delivery, year	31.6 ± 3.5	31.2 ± 4.1	0.679
Pre-pregnancy BMI, kg/m^2^	21.1 ± 3.0	21.4 ± 4.0	0.724
Gestational weight gain, kg	14.4 ± 4.3	14.6 ± 3.7	0.844
Gestational age, week	39.3 ± 1.1	39.3 ± 0.8	0.713
Serum vitamin D level, ng/mL	22.1 ± 4.5	33.2 ± 1.5	<0.001
Seasonal distribution of vitamin D measurement			0.920
Spring (March–May)	1 (1.69)	0	
Summer season (June–August)	30 (50.85)	13 (46.43)	
Autumn (September–November)	23 (38.98)	13 (46.43)	
Winter season (December–January)	5 (8.47)	2 (7.14)	
Delivery mode			0.991
Vaginal	38 (64.41)	18 (64.29)	
Cesarean	21 (35.59)	10 (35.71)	
GDM	24 (40.68)	11 (39.29)	0.902
HDP	6 (10.17)	1 (3.57)	0.421
**Infant characteristics**			
Sex, male/female	32/27	15/13	0.954
Birth weight, kg	3.2 ± 0.4	3.2 ± 0.5	0.939
Birth length, cm	49.9 ± 1.3	50.0 ± 1.9	0.969
Feeding practices			0.104
Breastfeeding	40 (67.80)	20 (71.43)	
Mixed	18 (30.51)	5 (17.86)	
Formula	1 (1.69)	3 (10.71)	
Days at collection of gut sample at 1 month	30.3 ± 2.9	31.1 ± 2.9	0.263
Weight at 1 month, kg	4.3 ± 0.5	4.4 ± 0.7	0.590
Length at 1 month, cm	54.9 ± 1.9	55.0 ± 2.2	0.899
Head circumference at 1 month, cm	36.8 ± 1.0	37.1 ± 1.3	0.316
Days at collection of gut sample at 6 months	186 ± 12.3	192 ± 12.2	0.063
Weight at 6 months, kg	8.0 ± 0.7	8.0 ± 1.0	0.991
Length at 6 months, cm	68.4 ± 2.3	68.4 ± 2.1	0.974
Head circumference at 6 months, cm	43.2 ± 1.2	43.4 ± 1.0	0.408

Data are shown as means (SD) or *n*(%). Significant differences in maternal and infant characteristics between the two groups are highlighted in bold. BMI, body mass index; GDM, gestational diabetes mellitus; HDP, hypertensive disorders of pregnancy.

### 3.2 Alpha and beta diversity

**Figure 1:**
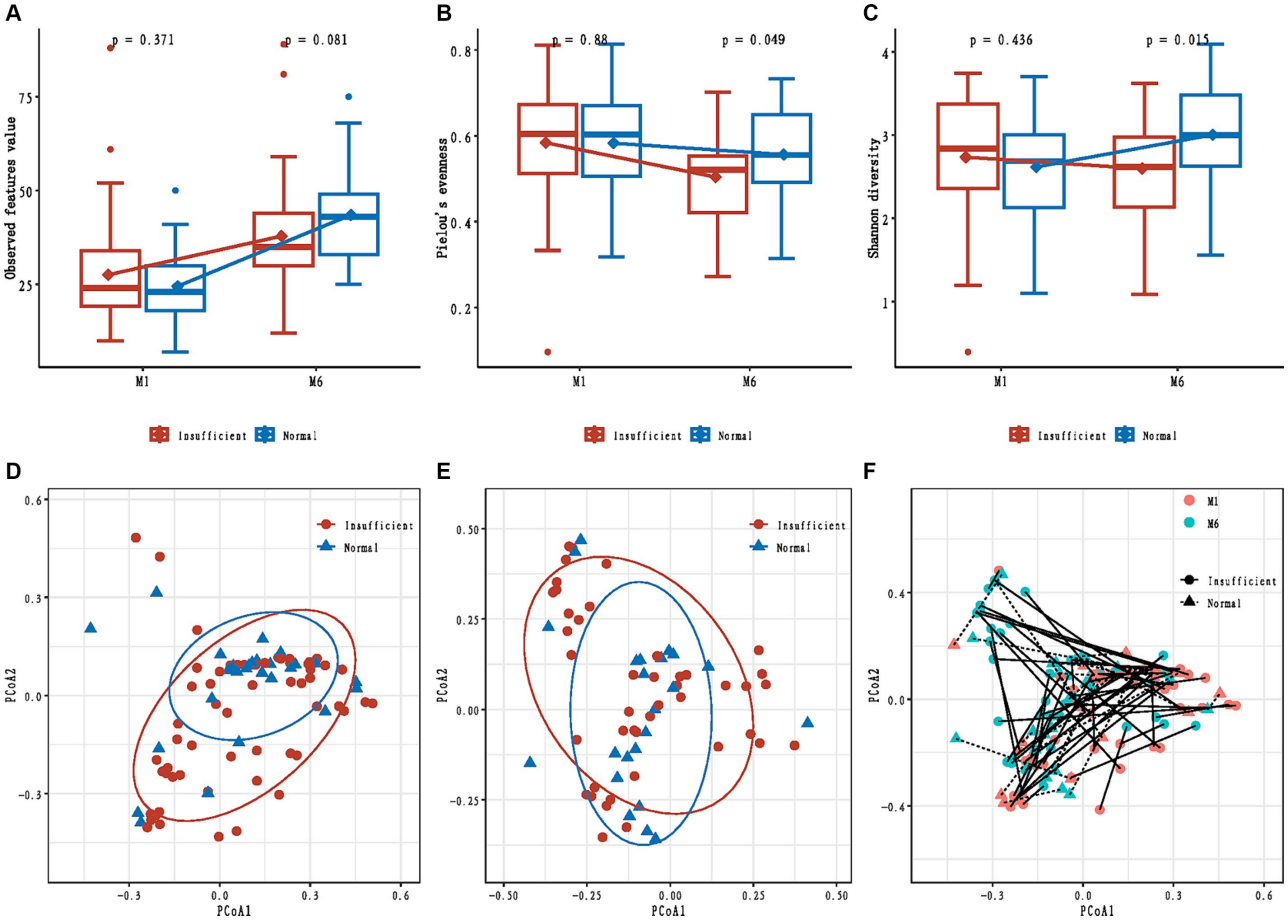
Alpha and beta diversity of infants’ gut microbiota in maternal vitamin D insufficient and normal group. **(A)** Comparisons of observed feature value; **(B)** Comparisons of Pielou’s evenness; **(C)** Comparisons of Shannon diversity; **(D)** Principal coordinates analysis based on Bray-Curtis distances in the M1 phase; **(E)** Principal coordinates analysis based on Bray-Curtis distances in the M6 phase; **(F)** Change in principal coordinates analysis based on Bray-Curtis distances from M1 to M6 phase.

In the M1 phase, the differences in alpha diversity indexes between the vitamin D insufficient group and normal group were not significant (all *p* > 0.05, Figures 1A–C); while in the M6 phase, compared to the normal group, the observed feature value was marginally lower (*p* = 0.081, Figure 1A), and the Pielou’s evenness and Shannon diversity were significantly lower in the insufficient group (*p* = 0.049 and *p* = 0.015, respectively, Figures 1B,C).

The mixed effect model analyses showed that the changes in observed feature value and Shannon diversity from the M1 to M6 phase were significantly different between the vitamin D insufficient group and normal group (*p* = 0.015 and *p* = 0.029, respectively).

We also tested if maternal vitamin D status during pregnancy was associated with the overall community structure and found no difference in Bray-Curtis distances between the two groups in both M1 and M6 phases (*R*^2^ = 0.015; *p* = 0.132, and *R*^2^ = 0.012; *p* = 0.713, respectively, Figures 1D,E). A significant temporal change in beta diversity was observed from the M1 to M6 phase (time: *p* = 0.032, Figure 1F); however, we found no differential change in beta diversity from the M1 to M6 phase according to maternal vitamin D status (time × maternal vitamin D status interaction: *p* = 0.768, Figure 1F).

### 3.3 Relative abundance

**Figure 2:**
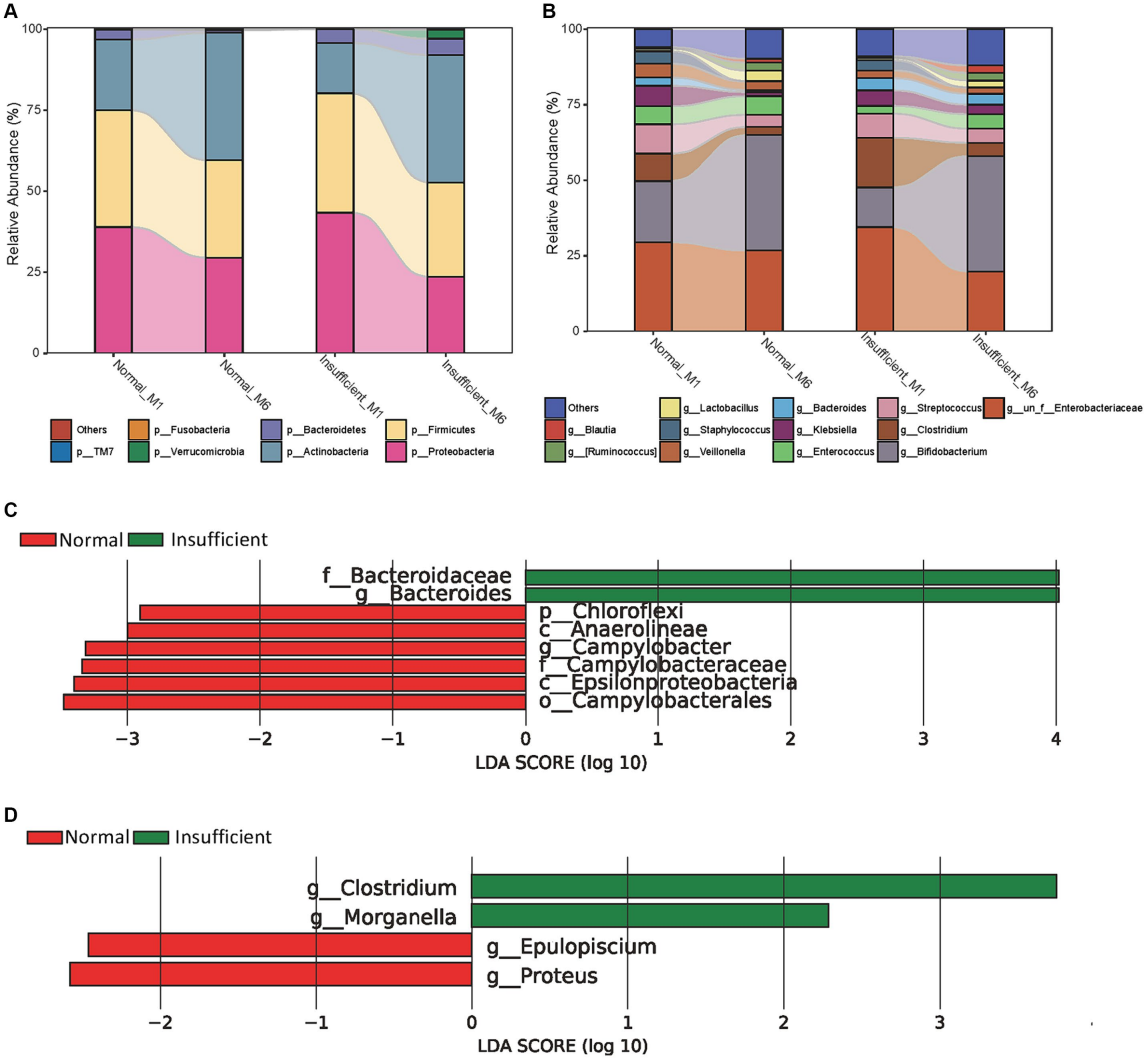
Relative proportions of abundant microbes and their differences between the vitamin D insufficient group and normal group. **(A)** Relative proportions of abundant microbes at the phylum level; **(B)** Relative proportions of abundant microbes at the genus level; **(C)** Histogram of LEfSe analysis in the M1 phase; **(D)** Histogram of LEfSe analysis in the M6 phase. The prefixes “p,” “c,” “o,” “f,” “g” represent the annotated level of phylum, class, order, family and genus.

Compositionally, the gut microbiota was dominated by members of the three major bacterial phyla *Proteobacteria*, *Firmicutes*, and *Actinobacteria*, accounting for approximately 95% in both the vitamin D insufficient and normal groups (Figure 2A). From the M1 to M6 phase, the relative abundance of phyla *Proteobacteria* and *Firmicutes* declined, while the phylum *Actinobacteria* rose. At the genus level, *Bifidobacterium*, *Clostridium*, *Streptococcus*, and one unclassified genus belonging to the family *Enterobacteriaceae* were the predominant genus, with genus *Bifidobacterium* rising and the other three genera declining in the M6 phase (Figure 2B). In addition, it is noteworthy that the relative abundance of the genus *Lactobacillus*, *Ruminococcus*, and *Blautia* also exhibited an upward trend from the M1 to M6 phase.

Using LDA, we identified the genus *Campylobacter* and all parent taxa within class *Epsilonproteobacteria* (i.e., family *Campylobacteraceae*, and order *Campylobacterales*), class *Anaerolineae* and its parent phylum *Chloroflexi* were depleted in the vitamin D insufficient group, while genus *Bacteroides* and its parent family *Bacteroidaceae* were enriched in the vitamin D insufficient group in the M1 phase (Figure 2C). In the M6 phase, we found genus *Clostridium* and *Morganella* were enriched, while genus *Epulopiscium* and *Proteus* were depleted in the vitamin D insufficient group (Figure 2D).

### 3.4 Abundant pathways

**Figure 3:**
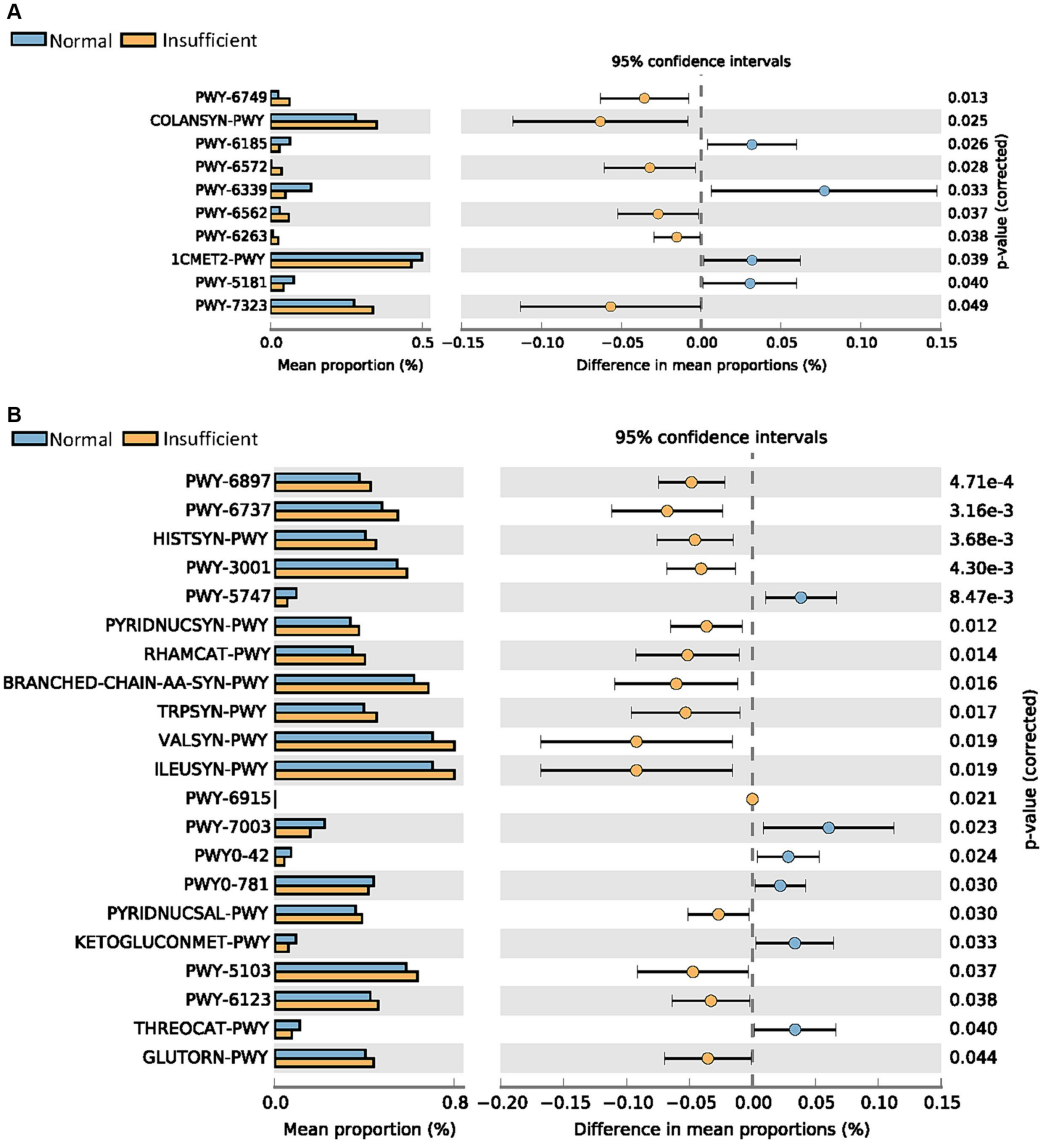
Comparisons of metabolic pathways between the vitamin D insufficient group and normal group in the M1 **(A)** and M6 **(B)** phases, respectively.

As shown in Figure 3, more pathways were significantly abundant in the vitamin D insufficient group, including PWY-6749 (CMP-legionaminate biosynthesis I), COLANSYN-PWY (colanic acid building blocks biosynthesis), PWY-6572 [chondroitin sulfate degradation I (bacterial)], PWY-6562 (norspermidine biosynthesis), PWY-6263 (superpathway of menaquinol-8 biosynthesis II), and PWY-7323 (superpathway of GDP-mannose-derived O-antigen building blocks biosynthesis) in the M1 phase, and quite a few pathways related to amino acid biosynthesis (HISTSYN-PWY, PWY-3001, BRANCHED-CHAIN-AA-SYN-PWY, TRPSYN-PWY, VALSYN-PWY, ILEUSYN-PWY, PWY-5103, GLUTORN-PWY), PWY-6737 (starch degradation V), and PWY-6123 (inosine-5′-phosphate biosynthesis I) in the M6 phase. In the normal group, we found that 1CMET2-PWY [folate transformations III (*E. coli*)] was enriched in the M1 phase, while 2-methylcitrate cycle I and II (PWY0-42 and PWY-5747) were enriched in the M6 phase.

### 3.5 Ecological association network

**Figure 4:**
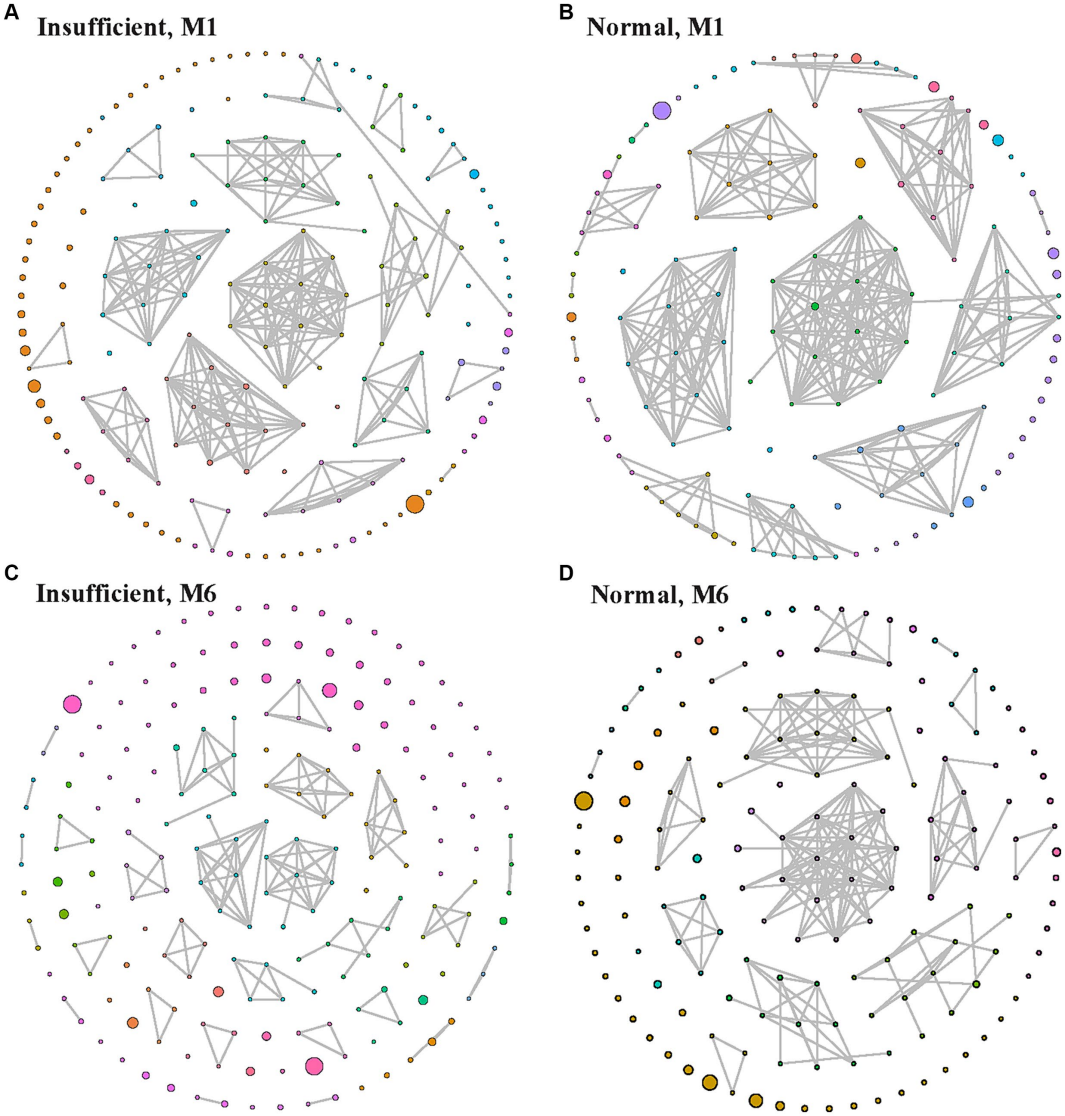
Ecological association network in the vitamin D insufficient group **(A,C)** and normal group **(B,D)** groups in the M1 **(A,B)** and M6 **(C,D)** phases, respectively. Each node indicates a genus, with larger node indicating higher relative abundance, and edges between nodes represent their predicted interactions. Different colors indicate different modularity classes.

Figure 4 shows the ecological association networks at the genus level in the vitamin D insufficient group and normal group in the M1 and M6 phases, respectively. In the M1 phase, the number of nodes and edges in the insufficient and normal groups looked similar (Figures 4A,B). However, in the M6 phase, the number of edges decreased dramatically, especially in the vitamin D insufficient group, indicating fewer microbial correlations in the vitamin D insufficient group.

## 4 Discussion

In the current study, we prospectively examined the influence of maternal vitamin D levels on the gut microbiota of the offspring during 1–6 months of life. We observed significantly lower Pielou’s evenness and Shannon diversity in the vitamin D insufficient group in the M6 phase, and the dynamic changes in alpha diversity from 1 to 6 months old were significantly different according to maternal vitamin D status. In terms of gut microbiota composition, there were significant differences between the vitamin D insufficient group and the normal group at both 1 and 6 months of age of infants. Moreover, among the predicted metagenome functions, pathways related to amino acid biosynthesis, starch degradation, and purine nucleotides biosynthesis were enriched in the vitamin D insufficient group. In addition, ecological network analysis showed an apparent decrease in microbial correlations from M1 to M6 phase, especially in the vitamin D insufficient group.

The role of vitamin D during pregnancy in the infant’s gut microbiota has been of increasing interest recently. Talsness et al. (11) found that maternal plasma vitamin D levels were negatively associated with counts of *Bifidobacterium* species and positively associated with *B. fragilis* counts in infants at 1 month of age. On the contrary, in our study, we found that the relative abundance of *Bifidobacterium* was lower and *Bacteroides* was higher in the vitamin D insufficient group in the M1 phase. Inconsistent results might be due to the different timing of vitamin D detection [i.e., the third (around 36 weeks) vs. second (15 ~ 19 weeks) trimester in the study of Talsness et al. (11) and our study, respectively] or racial differences (Dutch vs. Chinese). *Bifidobacterium*, also known as probiotics, is a natural part of the bacterial community in the human body and there is a symbiotic bacteria-host relationship with humans. *Bacteroides*, commonly found in the human gut, assist in breaking down food and producing valuable nutrients and energy that the body needs. Our study indicated that vitamin D insufficient during pregnancy may reduce the beneficial effect of *Bifidobacterium* on infants, but has an auspicious influence on the *Bacteroides* with a beneficial role.

In addition, we also found that the relative abundance of the genus *Bifidobacterium,* as well as *Lactobacillus*, *Ruminococcus*, and *Blautia,* exhibited an upward trend from M1 to M6 phase. *Lactobacillus* metabolizes carbohydrates to produce lactic acid, and there is strong evidence associating various *Lactobacillus* probiotics with several health benefits (23, 24). *Ruminococcus* obtains nutrients by breaking down cellulose that passes through the host’s digestive system. *Blautia* is a new functional genus with potential probiotic properties, such as biological transformation, regulating host health, and alleviating metabolic syndrome (25). From 1 month to 6 months old of infants, all these bacteria mentioned above were rising, suggesting that the gut microbiota in infants was developing toward a ever-risingly mature and beneficial direction over time.

Linear discriminant analyses showed that genus *Campylobacter* and its all parent taxa within class *Epsilonproteobacteria* were significantly depleted in the vitamin D insufficient group in the M1 phase. Such association was not observed in the M6 phase. *Campylobacter*, considered to be the most common bacterium that leads to human gastroenteritis, is one of the major causes of diarrhoeal diseases in the world, and it can be fatal among very young children (26). Our finding indicated that maternal vitamin D insufficiency during pregnancy might be a protective factor for infants at 1 month old. However, genus *Campylobacter* was only detected in a very small number of samples in the present study; therefore, the association between maternal vitamin D status and genus *Campylobacter* might be spurious. More researches are awaited in the future to confirm our findings.

In the M6 phase, genus *Clostridium* and *Morganella* were enriched, while genus *Epulopiscium* and *Proteus* were depleted in the vitamin D insufficient group. The identified association between the maternal vitamin D status and the relative abundance of genus *Clostridium* in offspring is in agreement with a previous finding that *Clostridium* was enriched in vitamin D receptor knockout mice (7). Besides, Chan et al. (27) found that vitamin D protected against *Clostridioides difficile* infection in mice by restoring macrophage lysosome acidification. *Clostridium* can be found in the human gastrointestinal tract, and its pathogenic species produce exotoxins that cause tissue and nerve necrosis, resulting in lesions. Previous studies have shown that colonization by *Clostridium* in the infant gut in early life was associated with increased risks of wheeze, eczema, and atopic dermatitis (28, 29). These findings suggested that maternal vitamin D insufficiency might be associated with higher levels of *Clostridium* in the gut of infants, which was linked to an increased risk of allergic diseases. Both *Morganella* and *Proteus* are opportunistic pathogens capable of causing major infectious disease problems (30). *Epulopiscium* is not usually found in the human gastrointestinal tract (31). Since the latter three were detected in only a few samples in our study, we would like not to discuss them here. More studies are needed in the future.

Through functional and pathway analysis, we found a large number of pathways enriched in the vitamin D insufficient group in the M6 phase, including multiple amino acid biosynthesis, starch degradation, and purine nucleotides biosynthesis pathways. Amino acids are the building blocks of proteins, which are of primary importance to the continuing functioning of life. Starch degradation is involved in energy metabolism, and nucleotides biosynthesis plays an essential role in DNA replication and transcription (32). These pathways enriched in the vitamin D insufficient group indicated that this group had more active microbial biosynthesis and more abundant microbial community metabolism. However, we do not know what kind of flora these abundant metabolic activities come from and whether it has a positive or negative impact on infant gut health.

Maternal vitamin D may potentially impact infant gut microbiome structure and composition via the following mechanisms. Ooi et al. (8) demonstrated that 1,25(OH)2D3 (i.e., active form of vitamin D) deficiency and vitamin D receptor knockout can affect the gut microbiome by increasing inflammation, and more gut inflammation provides a conducive environment for pathogens to proliferate at the expense of beneficial bacterial species. Vitamin D may also affect the gut microbiome by upregulating innate immunity, producing antimicrobial peptides through macrophages, maintaining the gut barrier function, and altering calcium and phosphate absorption (9). Since an infant’s gut microbiota inherited from the mother’s to some extent (33), therefore, we supposed that maternal vitamin D influenced maternal gut microbiota, and then be provisioned to fetuses *in utero*, which decided the first microbial colonizers of the infant’s gut. However, mechanistic links between maternal vitamin D status and gut microbiome in offspring remain to be elucidated.

The Developmental Origins of Health and Disease (DOHaD) theory believes that many diseases may originate from exposures *in utero* or during childhood. As a result, maternal vitamin D levels during pregnancy, as a modifiable factor, is of great public health significance in the prevention of many diseases, such as allergic diseases, by influencing the maintenance of gut microbiota homeostasis (10). Therefore, vitamin D supplementation is strongly recommended in clinic practice. However, due to the chronicity of vitamin D deficiency and the potential for physiologic adaptations to this condition, a longer supplementation period might be necessary, possibly even commencing prior to pregnancy (34).

Our study, to the best of our knowledge, was the first one to assess the influence of maternal vitamin D status during pregnancy on the gut microbiota in infants in a Chinese population, and newly reported that the dynamic changes of alpha diversity in infant’s gut microbiota from 1 to 6 months old were significantly different according to maternal vitamin D status using a prospective study design. Another advantage of this study was that the basic characteristics of the subjects in the vitamin D insufficient group and the control group were similar and comparable, which reduced the confounding of related factors.

One limitation of this study was the small sample size. However, given the sample size, standard deviation and difference in the present study, we would therefore calculated a statistical power of more than 85%, with a level of statistical significance of 5%; moreover, the basic characteristics were comparable in the two groups, strengthening the statistical power. Future studies require larger sample sizes, longer follow-up periods, and assessment of prenatal vitamin D at multiple time points. Secondly, the associations between maternal vitamin D supplementation during pregnancy and the infant’s gut microbiota were not studied in our research, since the vast majority of the included women were prescribed to receive a routine vitamin D supplementation of 125 IU per day, combined with calcium supplementation. In addition, unmeasured confounding factors such as maternal dietary habits, hygiene practices, and medication use may also affect infant gut microbiota structure and composition.

## 5 Conclusion

In brief, this study found evidence of an association between maternal vitamin D status with the gut microbiota structure and composition of infants in Shenzhen, China, notably a lower Pielou’s evenness and Shannon diversity, and a higher abundance of *Clostridium*, which might be linked to an increased risk of allergic diseases. Furthermore, function prediction showed that at 6 months of age, infants born to mothers with vitamin D insufficiency had more active biosynthesis and metabolism. These findings highlight that maternal vitamin D status played a pivotal role in shaping the early-life gut microbiota of the next generation. Therefore, it is of great significance to prevent vitamin D insufficiency in women, and policies or recommendations, such as routine vitamin D supplementation of 400 IU or more per day, and more outdoor activities to get adequate sun exposure during pregnancy, for the prevention of vitamin D insufficiency in China is highly essential.

## Data availability statement

The datasets presented in this study can be found in online repositories. The names of the repository/repositories and accession number(s) can be found here: https://db.cngb.org/, China National GeneBank DataBase.

## Ethics statement

The studies involving humans were approved by Ethics Committee of Shenzhen Baoan Women’s and Children’s Hospital. The studies were conducted in accordance with the local legislation and institutional requirements. Written informed consent for participation in this study was provided by the participants’ legal guardians/next of kin.

## Author contributions

QS: Conceptualization, Data curation, Formal analysis, Funding acquisition, Methodology, Visualization, Writing – original draft, Writing – review & editing. YL: Investigation, Validation, Writing – review & editing. TZ: Methodology, Visualization, Writing – review & editing. MX: Investigation, Validation, Writing – review & editing. BX: Formal analysis, Methodology, Visualization, Writing – review & editing. MW: Supervision, Writing – review & editing. YZ: Data curation, Funding acquisition, Project administration, Supervision, Writing – review & editing.
